# Access to Information and Degree of Community Awareness of Preventive Health Measures in the Face of COVID-19 in Spain

**DOI:** 10.3390/healthcare9020104

**Published:** 2021-01-20

**Authors:** Laura Gutiérrez-Velasco, Cristina Liébana-Presa, Elena Abella-Santos, Vega Villar-Suárez, Rocío Fernández-Gutiérrez, Elena Fernández-Martínez

**Affiliations:** 1 Complejo Asistencial Universitario de León (CAULE), 24071 Leon, Spain; lgutierrez@saludcastillayleon.es (L.G.-V.); rociofernandez@saludcastillayleon.es (R.F.-G.); 2 SALBIS Research Group, Faculty of Health Sciences, Universidad de Leon, Ponferrada, 24401 Leon, Spain; elena.fernandez@unileon.es; 3 Clara Isabel Santos García Pharmacy, 24010 Leon, Spain; eabella70@hotmail.com; 4 Institute of Biomedicine (IMBIOMED), Universidad de León, 24071 Leon, Spain; vega.villar@unileon.es

**Keywords:** COVID-19, coronavirus, preventive measures, information, community awareness, hand washing, mask, physical distance

## Abstract

The COVID-19 pandemic is posing a major health crisis. Spanish legislation establishes the mandatory use of masks and the implementation of hygienic measures such as hand washing and physical distancing. The aim of this study is to describe access to information and the level of community knowledge/adoption about the preventive measures proposed by the Spanish health authorities in response to the COVID-19 pandemic and to analyze the influence of socio-demographic factors in compliance among people over 18 years of age resident in Spain. An observational, descriptive and transversal study was conducted. Data was collected on sociodemographic variables, access to information and the degree of knowledge/adoption about the preventive measures: use of masks, hand hygiene and physical distancing. A total of 1811 people participated. The average age was 45.1 ± 15.1 years, predominantly female (69.3%), from an urban geographical area (74%), with a higher education level of 53.2%. Most of the respondents (57.5%) are or live with people at risk. The main access to information on preventive measures was from secondary sources (49.2%), with television being the main medium; 72.3% think that there are some difficulties in accessing information, while 8.7% of the participants do not consider the use of masks to be useful. As regards the choice of type of mask, the majority of people (44.8%) opt for the surgical variety; 88.5% of respondents believe that the physical distancing established is at least 1.5 m. This study confirmed that socio-demographic factors influence compliance with or the degree of knowledge/adoption of the preventive measures proposed to combat the COVID-19 pandemic and has made it possible to ascertain the sectors of the population with the greatest deficiencies in this respect. It shows the importance of implementing health information and education systems in the community, and it is advisable to promote specific programs aimed at men, people living in rural areas and people with a low level of education.

## 1. Introduction

The current COVID-19 pandemic is posing a major health crisis. The virus is highly infectious, caused by the new SARS-CoV-2 severe acute respiratory syndrome coronavirus, whose main transmission routes are respiratory droplets. While initially also targeting transmission by fomites [1], the most recent research states that it is rare under real conditions [2], and studies pointing to the airway as a mechanism of virus transmission are gaining more and more weight [3,4].

This new disease emerged in December 2019 in Wuhan (China) and rapidly spread throughout the world. In response to this global crisis, on 30 January 2020, the World Health Organization (WHO) qualified this situation as a public health emergency and on 11 March 2020 declared it an international pandemic [5].

On 31 January 2020, the first case of COVID-19 in Spain was officially identified, and on 14 March a Spanish state of alarm was declared [6], at which time a series of measures aimed at containing the SARS-Co-V2 virus were decreed by the competent bodies. On 20 June 2020, the state of alarm ended in Spain, and on 22 June the so-called new normality began throughout the country. In this new stage, the head of state legislated a series of measures to prevent the spread of the COVID-19. These regulations basically adopted basic and traditional public health principles regarding the transmission of infectious diseases, such as hand hygiene, the use of masks and physical distance. In Spain, following the publication of Order SND/422/2020 of 19 May [7], the legislation established the obligatory use of masks for all persons over six years of age, excluding some cases of illness or disability among others. Hygiene measures were also strengthened in all areas, highlighting the importance of hand washing and physical distance.

The use of masks makes it possible to limit the spread of viral respiratory diseases such as COVID-19. This measure was already used successfully in the control of the SARS epidemic in 2003 [8,9]. There are different factors to take into account when assessing the use of a mask. On the one hand, there are several types of masks on the market that have different filtration and protection characteristics. Those with the greatest filtering and protective capacity are the so-called surgical masks and personal protective equipment (PPE), and there are also categories within this classification. Both types are considered to be medical devices, so they comply with regulations and are designed, manufactured and evaluated in accordance with the relevant standards. On the other hand, there are hygienic masks, which include masks approved in accordance with a regulation, to fabric masks without proven effectiveness. It is important to highlight that several studies [10,11] have ensured that although hygienic masks offer fewer guarantees, even wearing a fabric mask increases protection as opposed to not wearing any type of mask at all.

Official bodies have drawn up recommendations as a guide to which mask is the most appropriate under the circumstances. According to a document prepared by the Ministry of Health and Consumer Affairs, the use of hygienic masks is recommended for healthy people, surgical masks for infected people (symptomatic or asymptomatic) and FFP2 PPE for those who care for or are in contact with infected people, including healthcare personnel [12]. Furthermore, it is important to consider the use that is made of this resource. Although facemasks have been shown to help control respiratory virus epidemics to some extent by being implemented as a single measure, their effectiveness is much greater when they are part of a series of measures that include physical distance and hand washing [13]. Likewise, it is essential to make proper use of them, as well as knowing the protocol for their placement and removal to avoid contamination derived from their handling, as recommended by the Ministry of Health in its document “The Correct Use of Masks” [14].

Hand washing is another important measure. The Spanish Ministry of Health and the WHO recommend that, for effective disinfection, hand washing should be carried out frequently, with water and soap or an alcohol-based solution, for between 40 and 60 s, and the procedure should include rubbing the palms and backs of the hands, the spaces between the fingers and the backs of the fingers [15,16,17].

Preventive measures are, so far, the only way of containing this pandemic, as there is still no effective treatment and there are many months left for a high percentage of the population to be properly vaccinated. Therefore, a high degree of public compliance with the proposed measures is crucial in containing the transmission of the virus. In this sense, various factors have been highlighted that influence adherence to these recommendations, such as risk perception, sociodemographic factors or the type of information received [18,19,20,21]. Information on the measures adopted is published in official media such as the Official State Gazette or the website of the Ministry of Health and Consumer Affairs and Social Welfare. However, these are not the only sources through which the citizen is informed nor are they the most demanded. Unofficial sources may issue unverified or untrue information, which may cause serious negative implications, in terms of prevention strategies, if this is prioritized over information based on scientific evidence [22].

Identifying possible population groups with ineffective access to information, or with insufficient knowledge/adoption about the preventive guidelines proposed, could highlight the need to implement health information and education systems on this subject. In this study, the following working hypothesis is put forward: “the socio-demographic factors that influence the compliance or degree of knowledge of the preventive measures on the part of the community with regard to the COVID-19 pandemic”, the null hypothesis being the independence of both variables. To this end, the objective is to describe access to information and the degree of community knowledge/adoption on the preventive measures proposed to combat the COVID-19 virus (use of masks, hand washing and physical distance) and to analyze the influence of socio-demographic factors on the degree of knowledge/adoption of these measures in the Spanish population.

## 2. Materials and Methods

This is a quantitative, observational, descriptive and transversal study.

### 2.1. Participants

The study population was made up of people over the age of 18 residing throughout Spain. By means of a non-probability convenience sampling, 1811 participants were selected. Two groups were differentiated within the sample: those who, through the questionnaire, stated to know or comply with the preventive measures recommended by the health authorities (group 1); and those who did not (group 2). The inclusion criterion for the group that did not comply with the preventive measures was that they had responded to the following items with the response indicated below:

- Item: “Under what circumstances do you think it is necessary to use a mask?”. Answer: “I do not think it is necessary as it does not fully protect”.
- Item: “Indicate what you usually wash your hands with”. Answer: “Only with water”.
- Item: “When I go out, to work, to a shop or a place of leisure, etc., if I don’t wear a mask and there is no protective element (for example, a screen)”. Answers: I should keep physical distance only with those people who have symptoms/If I am in an outdoor space, the measure of physical distance is not necessary, it is only for closed spaces.

### 2.2. Data Collection Instruments

The questionnaire used was prepared ad hoc for this study and was structured in three blocks. The first block included socio-demographic variables, where the following characteristics were collated: sex, age, geographical area, place of residence, level of study and belonging or being a relative of a risk group. The second block included three items relating to access to information on preventive measures against COVID-19. Data was collected on the media, the quality and quantity of information and the degree of accessibility to it. The third block was aimed at describing the degree of knowledge/adoption about the main preventive measures proposed by the health authorities: use of masks, hand hygiene and physical distance (see Table 1).

### 2.3. Procedure

The data from this study were collected online through Google Forms accessible at the following link: https://forms.gle/QrfGs4LvDTYVPGvc8. The questionnaire was disseminated on social networks during August and September 2020.

### 2.4. Statistical Analysis

The database and statistical analyses were carried out using SPSS 26.0 (Statistical Package for the Social Sciences) software. The analysis of the data was descriptive and analytical. Frequencies and percentages were used for the descriptive analyses and the Chi-square statistic for the bivariate analysis.

### 2.5. Ethical Considerations

Data collection was accompanied by a study information sheet, and written informed consent was obtained from all participants. Subjects voluntarily participated in the study, and their data were kept confidential and anonymous at all times. The data obtained from the research were treated in accordance with both the Constitutional Law 3/2018, of 5 December, on the Protection of Personal Data and the Guarantee of Digital Rights and the General Regulation on the Protection of Data of the European Union EU 2016/679 (RGPD), which came into force in Spain on 25 May 2018. This study was approved by the Ethics Committee of the University of León (ETICA-ULE-023-2020). The study carried out followed the standards set by the Declaration of Helsinki, complying with each of its basic principles.

## 3. Results

This study involved 1811 people from all the Spanish Autonomous Regions. The average age of the participants was 45.1 ± 15.1 years, with a range of 18 to 89 years and a homogeneous distribution in terms of age groups. Table 2 shows the socio-demographic characteristics of the sample: predominantly female (69.3%), urban geographical area (74%), higher education level (53.2%) and the majority of respondents (57.5%) are or live with people at risk (over 60 years old or with chronic pathologies).

**Figure 1:**
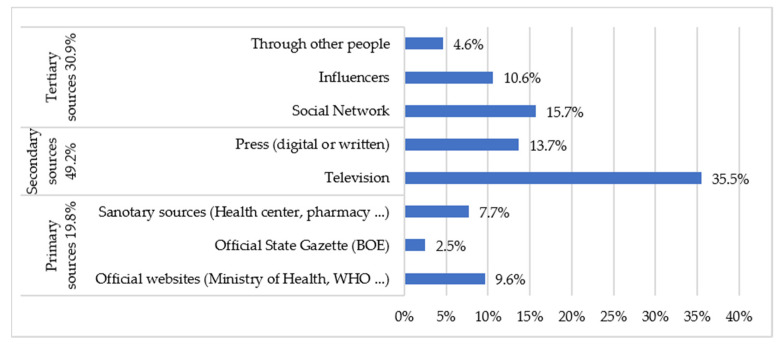
Information sources of preventive measures.

Firstly, with regard to access to information on preventive measures, Figure 1 shows the distribution of the different sources of information. The results indicate that the means of communication through which the most information on preventive measures (use of masks, hygiene measures, physical distance, etc.) was obtained was secondary sources (49.2%), with television being the main medium. For 30.9% of the sample, the channel of information on preventive measures was tertiary sources such as social networks, influencers or other people in the environment.

**Figure 2:**
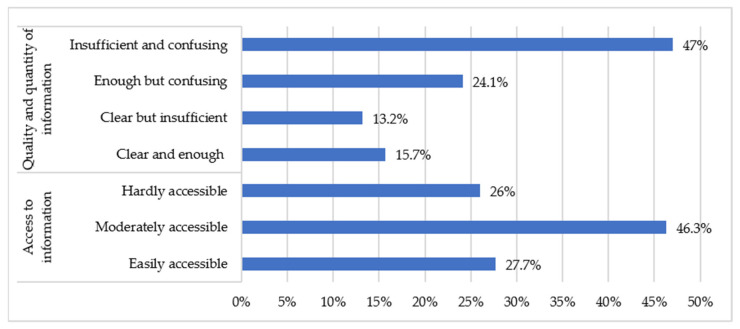
Quality, quantity and access to information on preventive measures.

Figure 2 shows that 15.7% of those surveyed considered the information provided by the competent health organizations (Ministry of Health, Ministry of Health and Consumer Affairs, World Health Organization) on preventive measures to be clear and sufficient. A total of 72.3% thought that there were some difficulties in accessing information.

**Figure 3:**
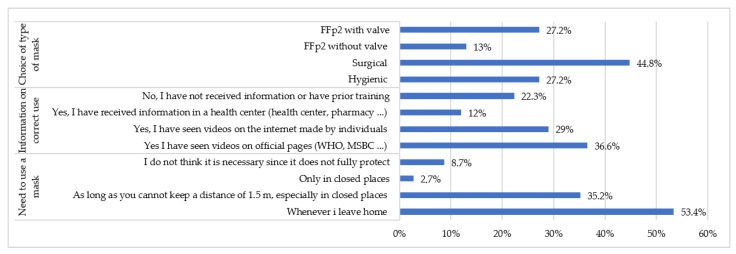
Use of the mask as a preventive measure.

Secondly, in relation to the degree of knowledge about preventive measures, Figure 3 shows the results regarding the use of masks; 8.7% of the participants did not consider the use of masks to be useful, because they believe that they do not protect against the COVID-19, 22.3% of the respondents received no information on the correct use of masks, and a significant number (29%) received information about masks through internet videos made by individuals. In terms of the choice of type of mask, the majority of people (44.8%) chose to wear a surgical mask for everyday situations; 27.2% of respondents said they used an FFp2 type mask with an exhalation valve.

**Figure 4:**
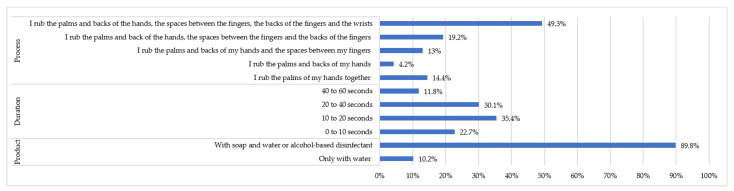
Hand washing as a preventive measure.

As for the preventive measure of hand washing, the vast majority (89.8%) used soap or an alcohol-based solution. These results can be seen in Figure 4. However, only 11.8% used the optimal time recommended by the health authorities for handwashing, although 41.9% spent an acceptable amount of time, and a little more than half of the people (50.7%) did not carry out an adequate handwashing regime.

**Figure 5:**
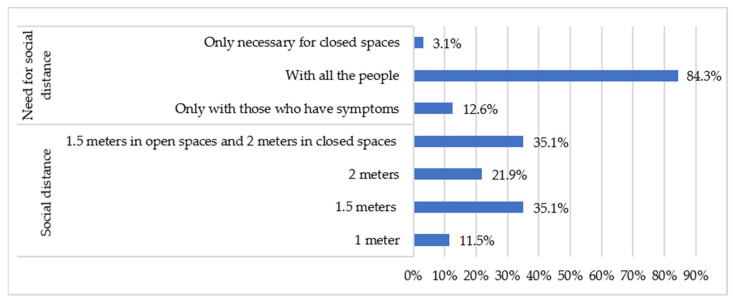
Physical distancing as a preventive measure.

The results show that 70.2% of the respondents thought that the preventive measure of the established physical distance was at least 1.5 m, and 12.6% considered this measure only relevant when they met people with symptoms of the COVID-19 (Figure 5).

According to the inclusion criteria described above, 92.5% of the participants (group 1) knew or met the preventive measures proposed by the health authorities, while 7.5% of the people (group 2) did not know or did not meet these preventive measures. As shown in Table 3, all calculated chi-square values were higher than the critical chi-square. The differences observed were not due to chance, and therefore the null hypothesis was rejected (the variables were independent). The alternative hypothesis was accepted, and it could be established that there is a relationship or dependency between the variables studied and the adoption of preventive measures by the community in relation to the COVID-19 pandemic.

**Figure 6:**
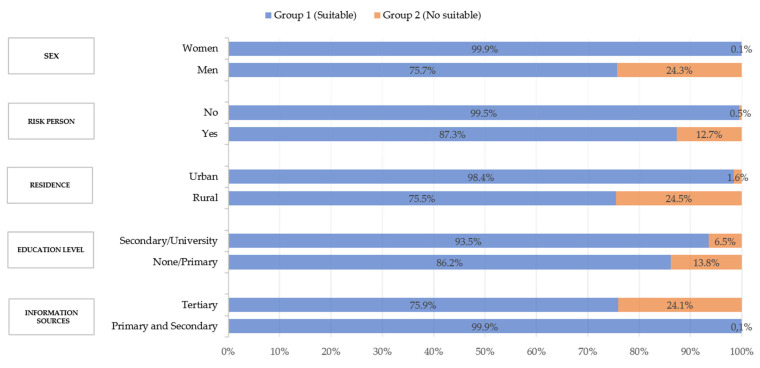
Relationship between socio-demographic variables and the degree of knowledge of preventive measures.

As shown in Figure 6, 24.3% of men reported not carrying out or not knowing the most basic measures recommended to avoid transmission of the virus, compared to 0.1% of women. The results observed in this analysis also showed that those respondents who are or live with people at risk adopted or knew better the measures proposed by the health authorities to stop the spread of the virus (99.5% compared to 87.3%). Furthermore, a marked difference was found in relation to the geographical area; in rural areas they adopted preventive measures less (24.5%) than those living in urban areas (1.6%). Participants with a higher level of education showed better data on knowledge and compliance with the proposed measures (93.5% compared to 86.2%), and 99.9% of people who said they obtained information on preventive measures through reliable sources (primary and secondary) as the main means were those who had a greater degree of knowledge or compliance with them (99.9% compared to 75.9%).

## 4. Discussion

This study attempts to identify possible population groups with ineffective access to information, or with an insufficient level of knowledge about the preventive guidelines proposed against COVID-19 by the Spanish Government on May 2020, as well as their degree of compliance with these measures. The information received about the different aspects of the pandemic can be an important determinant of how the population responds to it and, therefore, influence its evolution.

The results of our study show that almost half of the respondents choose secondary sources to inform themselves, mainly television. However, a considerable (30.9%) percentage of the sample relies on tertiary sources such as social networks, influencers or other people in the environment as their channel of information on preventive measures. These sources of information may contain biased or uncertain information, as shown in a study carried out by Islam et al. [22], in which 2311 articles published on social networks from 87 different countries were evaluated; 89% were classified as rumors or unverified information, including conspiracy theories about the origins of the pandemic with a malicious intent (7.8%) or discriminatory content related to the COVID-19 (3.5%). Spain was among the four countries where most cases of this type of misinformation occurred. This situation leads to a high risk of generating confusion in society regarding the prevention of COVID-19.

On the other hand, a study carried out in 2020 [19] showed that 84% of respondents considered health care providers to be a very reliable source of information, yet only 5% said that they were the most consulted means of obtaining information about the pandemic. This figure is close to 7.7% in this study.

One of the factors that can influence the choice of information source can be its accessibility to all sectors of the population. Of the people surveyed for this work, 72.3% found it difficult to access information from official bodies, while only 15.7% considered this information clear and sufficient. In Italy, only 58% of the population studied considered the information provided by the government to be true and appropriate, according to work carried out by Barari et al. [23]. It is likely that, in this sense, the various changes that have taken place in the discourse of the different authorities on recommendations on personal protection measures will have an influence. These modifications have been caused by the initial uncertainty about the knowledge of the virus and by the initial use of inconclusive research.

The vast majority of people participating in this study understand the need to use a mask as a measure against the COVID-19 virus. However, 8.7% of people do not consider masks as useful because they believe that they do not protect against the COVID-19. The data obtained also show the diversity in the type of mask used by the population in everyday situations. Although in healthy people wearing a hygienic mask would be sufficient, a surgical mask and FFp2 without exhalation valve options could be accepted as valid, as they would all prevent transmission from the mask wearer to the environment. There is currently a controversial debate about the use of masks as a means of limiting the spread of the disease. While there are different studies [11,24,25] that endorse this intervention as an effective and valuable measure, various investigations coexist [26,27] that have shown lack of applicability or efficacy of masks against COVID-19.

This is not the case with the FFp2 mask with exhalation valve, which was chosen by 27.2% of participants. Although it does protect against transmission from the environment to the mask wearer, it does not do so in the opposite direction. In other words, if this type of mask were to be used by a person carrying the virus, who is asymptomatic and therefore believed to be healthy, it would not prevent the contamination of his/her environment. This poses a risk of exposure for people wearing surgical or hygienic masks who come into contact with individuals wearing masks with exhalation valves. Since its use is not recommended and even prohibited in some regions [28,29], with the exception of some professionals, special attention should be paid to this sector of the population, and control measures should be adopted in their distribution and use, as they can pose a silent risk due to the false perception of security they offer.

As far as the extent of hand washing is concerned, the vast majority of society follows the recommendations of the Ministry of Health and the WHO insofar as they use soap or an alcohol-based solution. However, in terms of time and procedure, there are shortcomings. Here, health education to the community by the health workers is crucial to achieve convenient and appropriate levels of hygiene as a preventive means of stopping the transmission of the virus [30].

There is evidence to suggest that society is confused about the physical distance established by the authorities; however, it is clear that the vast majority of people are aware of this measure and apply it as the authorities have proposed. The study by Barari et al. (2020) shows that most people believe that the measure of physical distance is appropriate, including quarantine, and that they are willing to comply with it. In this case, the focus should be on those people (12.6%) who see this measure as only relevant to those with symptoms of the COVID-19, as recent studies [31] have determined that asymptomatic persons infected with SARS-CoV-2 are potentially transmitting the virus.

A clear influence of sex was found in the degree of knowledge or compliance with the measures. This data is consistent with information provided in other studies that have associated women with a higher degree of compliance with preventive measures [19], or indicated that women are more likely to follow COVID-19 guidelines [32].

According to the results observed, people who are or live with people at risk take or know better the appropriate measures to stop the contagion. This data is transcendental considering the discrepancies in the prognosis of the COVID-19 a priori in older people or those with previous pathologies [33]. Previous studies [18] have determined that the population over 70 years of age is where preventive measures have been most easily introduced. This statement is in line with the data in this study, since this risk group includes people over 60 years of age or with chronic pathologies. Other studies have linked the perception of risk or the degree of anxiety caused by the situation of uncertainty generated by the pandemic with greater compliance with the established measures [19,21,34] and. have determined that the population over 70 years of age is where preventive measures have been most easily introduced. This statement is in line with the data of this study, since this risk group includes people over 60 years of age or with chronic pathologies. Other studies have linked the perception of risk or the degree of anxiety caused by the situation of uncertainty generated by the pandemic with greater compliance with the established measures.

In the study by Kwok et al. [19] It was also observed that place of residence could be another factor influencing the sense of risk and a better understanding of the need to comply with preventive measures. This is the case in the New Territories in Hong Kong where there is a greater accumulation of inhabitants and more traffic compared to other areas of Hong Kong. Our study shows consistent results when comparing compliance with measures in rural areas (24.5%), where nuclei are composed of fewer inhabitants, with respect to urban areas (1.6%), where the accumulation of people and traffic is greater.

The data shown in the relationship between the sources of information and the degree of knowledge or compliance with preventive measures are relevant. The people who claim to obtain information on preventive measures through reliable sources as their main means are those who have a greater degree of knowledge or compliance with them. The fact that only a small part of the population (15.7%) considers the official information disseminated to be clear and sufficient and that there is a greater degree of compliance with the proposed measures among groups with a higher level of education (93.5%) makes us see the necessity of promoting primary information sources and in a different format that is easily understood by the whole population. Health centers provide reliable information and are easily accessible to the whole community [35]. Therefore, they are proposed as appropriate means to provide health education on preventive measures against COVID-19.

This study has limitations. The main limitation is that it uses a transversal design that cannot indicate causality. In future studies it would be useful to carry out longitudinal designs that can indicate how these variables behave over time and be able to carry out causal explanations as well as experimental designs where health education interventions are proposed to population groups. Another limitation refers to the collection of data, which was carried out by means of self-administered questionnaires, so that the biases of subjectivity and reliability are present. The technological tools through which the questionnaire was disseminated and the data collected were able to condition the subjects who answered, not reaching the entire population. In future studies, different data collection strategies can be used to access the population of different groups.

## 5. Conclusions

This study confirmed that socio-demographic factors influence compliance with or the degree of knowledge of the preventive measures proposed to combat COVID-19 and has made it possible to ascertain the sectors of the population with the greatest deficiencies in this respect. It shows the relevance of implementing health information and education systems in the community, it being more effective to emphasize with specific programs oriented to these community subgroups (men, rural areas, low level of studies and people who receive information mainly through tertiary sources).

With the necessary resources and through the family and community care of the Spanish health system, it would be possible to design and implement health education programs (hand washing, physical distance and the use of a mask) that promote greater knowledge and adherence to the preventive measures against COVID-19.

It would be interesting to implement coordinated public information campaigns that reinforce key messages to shape people’s behavior and prevent the spread of the virus, influencing the knowledge and willingness of the population to comply with health measures that aim to limit transmission of COVID-19. In addition to conventional media, social networks and television should also be included (as this study has shown), as they are essential to help spread this information to a large audience.

## Figures and Tables

**Table 1:** Data collection questionnaire on access to information and the degree of community awareness of preventive measures in the face of the COVID-19 in the Spanish population.

**Block II. Access to Information on Preventive Measures**		
By what means of communication have you received more information about the preventive measures established in the new normality (use of masks, hygiene measures, physical distance, etc.)?		Television press (digital or written). Social networks. Influencers. Through other people. Official websites (Ministry of Health, WHO, etc.). B.O.E. Sanitary (health center, pharmacy, etc.).
How do you consider the information provided by the competent bodies in the field of health (Ministry of Health, Ministry of Health and Consumer Affairs, World Health Organization) on the preventive measures established in the new normality?		Clear and sufficient. Clear but insufficient. Sufficient but confusing. Insufficient and confusing.
It considers that the information provided by the competent bodies in the field of health (Department of Health, Ministry of Health, World Health Organization) on preventive measures is:		Easily accessible. Moderately accessible. Difficult to access.
**Block III. Degree of Knowledge/Adoption of Preventive Measures**		
Use of Masks	In what circumstances do you think it is necessary to use a mask?	Whenever you leave the house. Whenever you can’t keep a distance of 1.5 m, especially indoors. Only in closed places. I do not think it is necessary since it does not fully protect.
	Have you been informed about the correct way to put on and take off your mask?	Yes, I have seen videos on the websites of official organizations (Department of Health, Ministry of Health, WHO, etc.). Yes, I have seen videos on the internet made by individuals. Yes, I have received information at a health center: hospital, primary care center, pharmacy. No, I have not received any information about it and I do not have any previous training.
	Which mask do you think a person without risk factors should wear to the supermarket?	Hygienic. Surgical. FFp2 with valve. FFp2 without valve.
Hand Washing	Indicate what you usually wash your hands with:	With water only. With soap and water or alcohol-based disinfectant.
	How much time do you spend washing your hands?	From 0 to 10 s. From 10 to 20 s. From 20 to 40 s. From 40 to 60 s.
	Please indicate which of the following procedures best suits your regular hand washing:	I rub my palms together. I rub my palms and the backs of my hands. I rub the palms and backs of my hands and the spaces between my fingers. I rub the palms and backs of the hands, the spaces between the fingers and the backs of the fingers. I rub the palms and backs of the hands, the spaces between the fingers, the backs of the fingers and the wrists.
Physical Distancing	The physical distance in the “new normal” to prevent the spread of COVID-19 is:	1 m. 1.5 m. 2 m. 1.5 m in open spaces and 2 m in closed spaces.
	When I go out, to work, to a shop or a place of leisure, etc., if I don’t wear a mask and there isn’t some kind of protective element (for example, a screen):	I should keep physical distance only from those people who have symptoms. I have to keep my physical distance from everyone. If I am in an open space, the measure of physical distance is not necessary, it is only for closed spaces.

**Table 2:** Descriptive statistics of the sample.

Socio-Demographics Variables		*N* (%)
Sex	Men	556 (30.7%)
	Women	1.255 (69.3%)
Age	Young adults (18–35)	489 (27%)
	Mature adults (36–59)	652 (36%)
	Seniors (≥60)	670 (37%)
Residence	Rural	1.340 (74%)
	Urban	471 (26%)
Educational level	None	51 (2.8%)
	Primary	203 (11.2%)
	Secondary	594 (32.8%)
	University	963 (53.2%)
Person or cohabiting person of risk	No	770 (42.50%)
	Yes	1.041 (57.50%)
Autonomous Region	Andalucía	55 (3%)
	Aragón	76 (4.2%)
	Asturias	53 (2.9%)
	Canarias	83 (4.6%)
	Cantabria	26 (1.4%)
	Castilla y León	681 (37.6%)
	Castilla la Mancha	47 (2.6%)
	Cataluña	73 (4%)
	Extremadura	60 (3.3%)
	Galicia	60 (3.3%)
	Islas Baleares	60 (3.3%)
	La Rioja	56 (3.1%)
	Madrid	282 (15.6%)
	Murcia	54 (3%)
	Navarra	45 (2.5%)
	The Basque Country	64 (3.5%)
	Valencia	36 (2%)

**Table 3:** Descriptive statistics and chi-square test for the variables studied.

Socio-Demographic Variables	Chi-Square ^1^
Sex	255.5
Person or cohabiting person of risk	304.1
Residence	310.5
Educational level	271.1
Information sources	290.4

^1^ The critical chi-square for a significance level of 0.05 and a degree of freedom of 1 was 3.8415.

## Data Availability

Not applicable.
